# Retraction: Adherence towards COVID-19 mitigation measures and its associated factors among Gondar City residents: A community-based cross-sectional study in Northwest Ethiopia

**DOI:** 10.1371/journal.pone.0347170

**Published:** 2026-04-13

**Authors:** The PLOS One Editors

This article [1,2] was identified as one of a series of submissions for which we have concerns about authorship and adherence to the journal’s first and second publication criteria.

In addition, the article [1] reports the same study and data set as a previously published article [3, retracted in 4] that was not cited or discussed in [1] and an article published later by the same author group [5, retracted in 6].

In light of these issues, the *PLOS One* Editors retract this article.

ZNA did not agree with the retraction. MWM, AGM, DMG, GMK, MKY, SYT, KAG, HSM, AWA, CAW, GMB, NTA, CDA, TA, ATT, BKR, EBT, AAT, ZA, HD, KTG, GGK, THM, SD, JA, TA, and MA either did not respond directly or could not be reached.
